# Biosecurity in Village and Other Free-Range Poultry—Trying to Square the Circle?

**DOI:** 10.3389/fvets.2021.678419

**Published:** 2021-06-02

**Authors:** Joachim Otte, Jonathan Rushton, Elpidius Rukambile, Robyn G. Alders

**Affiliations:** ^1^Berkeley Economic Advising and Research, Berkeley, CA, United States; ^2^Institute of Infection, Veterinary and Ecological Sciences, University of Liverpool, Liverpool, United Kingdom; ^3^Quality Assurance Unit, Tanzania Veterinary Laboratory Agency, Dar es Salaam, Tanzania; ^4^Kyeema Foundation, Brisbane, QLD, Australia; ^5^Development Policy Centre, Australian National University, Canberra, ACT, Australia

**Keywords:** poultry, village, biosecurity, risk, economics, household

## Abstract

Village poultry commonly suffer significant disease related losses and a plethora of biosecurity measures is widely advocated as a means to reduce morbidity and mortality. This paper uses a household economy perspective to assess some “economic” considerations determining biosecurity investments of village poultry keepers. It draws on the 2012/13 Tanzania National Panel Survey (TZ-NPS), which covered 1,228 poultry-keeping households. Disease was the most frequently reported cause of bird losses and, in the majority of households, accounted for more than half of reported bird losses. However, given that poultry rarely contributed more than 10% to total annual household income, for 95% of households the value of birds lost to disease represented <10% of annual income. The value placed on poultry within households may vary by gender and the overall figure may mask differential intra-household impacts. The break-even cost for various levels of reduction of disease losses is estimated using a partial budget analysis. Even if achieved at no cost, a 75% reduction in disease-associated mortality would only result in a one percent increase of annual household income. Thus, to the “average” village poultry-keeping household, investments in poultry may not be of high priority, even when cost-effective. Where risks of disease spread impact on the wider community and generate significant externalities, poultry keepers must be supported by wider societal actions rather than being expected to invest in biosecurity for purely personal gain.

## Introduction

Domesticated animals^1^ deliver significant monetary and non-monetary products and services to society. These benefits can be put at risk by infectious and parasitic diseases, which can have a dramatic impact on productivity through morbidity and mortality and hence directly and indirectly affect the associated human communities. The generic recommendation that livestock keepers “enhance biosecurity” is a widely proposed solution to the threat of animal disease in the livestock development literature. FAO (1) defines biosecurity as “*the implementation of measures that reduce the risk of the introduction and spread of disease agents*,” comprising three principal elements: (i) segregation, (ii) cleaning, and (iii) disinfection.

“Improved biosecurity” is said to increase productivity, enhance income and food security, protect human health, and reduce antimicrobial use [e.g., (2–4)]. The recommendation to enhance biosecurity is not only leveled at market-oriented/commercial livestock producers but also at low-input low-output livestock keepers, many of which keep small flocks of free-range/scavenging poultry [see (5) for a review of pertinent literature].

Rural, extensive poultry raising (“village poultry”) is extremely popular in low- and middle-income countries (LMICs), because it does not need a large investment, poultry reproduce rapidly, and birds can scavenge for feed. They thrive on kitchen waste, broken grains, earthworms, snails, insects and vegetation. Village poultry make a significant contribution to poverty alleviation and household food and nutrition security by providing scarce animal protein and bioavailable micronutrients in the form of meat and eggs and income to meet essential family needs (6, 7). In many LMICs village poultry remain by far the most numerous type of poultry raised and, despite small flock sizes, in aggregate account for 60–90% of the poultry population (8). In Indonesia for instance, 22 million households raise village chickens of which only 1 million (<5%) have more than 30 birds (9).

A wide array of recommended biosecurity measures can be found in the literature, yet no standardized classification exists. Table 1 lists the main biosecurity recommendations for backyard/village poultry compiled by Conan et al. (5) in their systematic review of biosecurity measures for backyard poultry.

**Table 1:** Recommended biosecurity measures for backyard/village poultry [elaborated from Conan et al. (5)], rationale, cost and comment.

**Measure**	**Rationale**	**Cost**	**Comment**
**Structural**			
Indoor raising	Limits contacts with wild birds, other flocks and people outside the household	Building, feed, litter and additional labor, possibly different disease profile leading to requirement of additional medicines and skills	Defeats the entire rationale of backyard poultry keeping. If sheds are open (likely to be the case in tropical climates) there will still be contact with wild birds.
Fences to limit free-ranging	Limits contacts with flocks and people outside the household	Cost of fencing plus required extra feed	Contact with wild birds and pests may be reduced, but may increase as feed can attract wild birds and pests
**Structural and/or operational**			
Separation by age (and poultry species)	Reduces passing on of infection from older to younger birds or between poultry species	Requires some form of “fencing”/physical barrier and restricting access to the area	If birds scavenge, they will still be exposed to wild animals and pests. Single age flocks are more susceptible to morbidity during outbreaks of diseases such as infectious bursal disease.
Quarantine of introduced birds for 14 days	Reduces risk of exposure to pathogens possibly carried by introduced birds	Requires “quarantine pen/area” and probably additional feed and labor	May facilitate theft of birds quarantined away from homesteads.
Separation of sick birds	Reduces exposure to pathogen responsible for disease	Requires “quarantine pen/area” and probably additional feed and labor	Birds can be infective before showing signs of disease and measure should be accompanied by cleaning and disinfection
**Operational with additional expense (incl. family labor)**			
Cleaning and disinfection	Reduces pathogen load in the house/pen and on equipment	Cost of detergent/disinfectant and (family) labor	Reduces within-flock spread but not introduction
Cleaning of food and water containers	Reduces pathogen load in feed and water	Cost of sanitizers and (family) labor	Reduces within-flock spread but not introduction
Secure safe water	A number of poultry diseases can be transmitted by drinking water.	Cost of disinfectant and (family) labor	No specific advice on how. Even tap water may not be safe in many LMIC locations
Composting manure outside flock area	Inactivates pathogens that are excreted with poultry feces.	Compost bin, labor required to collect manure (and bedding) and to manage composting process	Only feasible where/when birds are kept in a circumscribed area, e.g., night pen. Reduces within-flock spread but not introduction.
Early removal and adequate disposal of dead birds	Reduces exposure to pathogen responsible for disease	Labor required to regularly check the flock; disposal can be perceived by vulnerable households as a loss of scarce food	Reduces within-flock spread but not introduction.
Vaccination^1^ against endemic diseases of importance	Reduces poultry morbidity/mortality and replication and spread of infectious agents	Vaccine, vaccinator fee	Does not reduce risk of pathogen introduction, is pathogen specific, may not protect against infection, may give false sense of security
**Operational, no apparent additional expenses (but opportunity costs)**			
Source poultry from trusted/disease-free flocks	Reduces likelihood of introducing pathogen via incubating or healthy carrier		Actually quite frequently practiced (possibly even the norm) as markets are often distant and birds can easily be sourced from “trusted” neighbors. Disease freedom is difficult to ensure given the limited testing capacity in LMIC.
Avoidance of live bird markets and other farms	Reduces risk of introducing pathogen on shoes, clothes, hands of poultry keeper		Given small number of birds sold/bought (possibly mostly at farm gate), visits to live bird markets may not be particularly frequent.
Visitor restriction	Reduces risk of introducing pathogen on shoes, clothes, hands of visitor		

1 *Not included in Conan et al. (5)*.

The principles of biosecurity are well-defined and practical measures have been devised, yet, with the exception of vaccination against selected diseases, the authors have been unable to find scientific evaluations of the “protective” effect for specific measures in commercial, let alone backyard poultry. Even less information on the benefit-cost ratio of specific biosecurity measures for individual poultry keepers has been generated. In fact, Conan et al. (5) conclude: “*We are left with the impression that the proposed lists of recommendations were made without weighing biosecurity measures according to prioritization criteria, efficiency or financial and technical feasibility*.”

The technocratic view of many development practitioners is, that farmers “do what they do, because they do not know better,” i.e., lack technical knowledge. However, farmers operate in economic, social and ecological contexts and their behavior may well be “rational” if these contexts are better taken into account. In order to address the gap in information identified, the paper presents a generic assessment of the “economic” aspects determining biosecurity investments of village poultry keepers by adopting a household economy perspective. It draws on the 2012/13 Tanzania National Panel Survey covering 1,228 poultry keeping households and is structured as follows (i) a description of village poultry keeping in Tanzania (ii) an analysis of poultry losses, the role of diseases, and the magnitude of losses in relation to total household income, (iii) a partial budget analysis of break-even cost of theoretical biosecurity investments leading to reduction of (observed) disease losses by 10, 25, 50, and 75%, and (iv) discussion and conclusions.

## Key Characteristics of Village Poultry and their Role in the Household Economy in Rural Tanzania

The following description of village poultry keeping in Tanzania is based on data collected by the Tanzania National Bureau of Statistics (TZ-NBS) as part of the implementation of the 2012/13 Tanzania National Panel Survey (TZ-NPS) (10). The TZ-NPS includes an expanded livestock module, with between 80 and 100 questions. In comparison, traditional living standards measurement surveys (LSMSs) include between 5 and 20 questions on livestock. In addition to LSMS information items, the TZ-NPSs collect information on (i) livestock ownership and herd/flock dynamics (e.g., sales, thefts, gifts, etc.), breeds kept, differentiated as local/indigenous vs. improved/exotic; (ii) use of inputs, including feed, water, labor, vaccines and drugs; (iii) production and use of livestock products and services, such as meat, milk and eggs, but also dung and traction; and (iv) sale and home-consumption of animal source foods.

The 2012/13 TZ-NPS collected data from 3,154 randomly selected rural households. Of these, 1,751 (56%) owned livestock, 1,228 (39%) owned poultry and 495 (16%) owned poultry as their sole type of livestock. Mean and median flock size of poultry owning households was 13.1 and 10.0 birds, respectively. Fifty-six percent of flocks consisted of 10 birds or less, 43% of flocks fell into the range of 11–50 birds and only 13 flocks (≈1%) had more than 50 birds (excluded from further analysis).

The vast majority of birds were of indigenous breed (15,036 vs. 27 “exotic”) and flocks were self-replacing. Over the recall period of 1 year, 95% (19,743/20,780) of recorded “entries” were hatched within the flock. Seventy percent of flocks did not have a bird added from “outside” and the 30% of flocks that introduced birds, either through purchase, as gift or payment, introduced a median number of three birds.

Birds from 22% of the households scavenged exclusively while those from 73% of the households were supplemented with some household “waste.” Only 5% of households provided small amounts of other feed (not further specified). None of the flocks were housed during daytime while 80% of households kept their birds indoors at night, either in chicken coop (46%) or in the family house (34%). Annual expenditure on poultry was low, with 77% of households having spent nothing on their poultry over 12 months.

Seventy-three percent of households had slaughtered (for home consumption) and 40% had sold birds over the past year. The average number of birds consumed (across all households) was 2.9 (median 2) and the average number of birds sold was 2.2 (median 0). Only 8% of households had sold eggs in the past 12 months and, overall, around 95% of eggs produced remained in the household. The dataset does not provide information on the numbers/proportions of eggs used for hatching and home consumption.

The average annual household income of all rural households keeping poultry was 2.3 million (median 1.6 million, IQR 0.9–3.0 million) TZ Sh. (app. USD 1,480) (Table 2). Crop production contributed the largest average within-household share of income (52%) followed by non-agricultural activities (34%) while income from livestock (poultry and other species) contributed an average share of 14%. Compared with households keeping poultry and other types of livestock, households with poultry as their only type of livestock averaged a slightly lower annual income of 2.0 million TZ Sh. (median 1.3 million, IQR 0.7–2.4 million), with crops, non-agricultural activities and poultry contributing 52, 40, and 8%, respectively.

**Table 2:** Mean and median annual income (thousand TZ Sh.^1^) of rural poultry-keeping households (hhs) in Tanzania in 2012/2013 by income source.

	**All poultry keeping**			**Poultry only livestock**		
	**hhs (1,214^2^)**			**hhs (495)**		
	**Mean**	**Median**	**Ratio^3^**	**Mean**	**Median**	**Ratio^3^**
Total	2,319	1,619	1.43	1,976	1,330	1.49
Crops	984	758	1.30	765	614	1.25
Livestock	314	52	6.03	119	12	9.56
Non-ag.	1,019	255	3.99	1,092	282	3.87

1 *1 USD ≈ 1,570 TZ Sh*.

2 *No income data for one household*.

3 *Mean/median*.

As indicated by the high mean-to-median ratio, income from livestock/poultry was extremely skewed (11) with a small number of households obtaining a relatively high income from livestock. Given total income is much more evenly distributed than livestock income, it follows that some households receive a large share of their income from livestock/poultry. Table 3 presents the distribution of share of household income from livestock/poultry of rural poultry-keeping households. Distribution of the control of household income could not be disaggregated by gender.

**Table 3:** Distribution of share (%) of household income from livestock/poultry of rural poultry-keeping households (hhs) in Tanzania in 2012/2013.

	**All poultry keeping**		**Poultry only livestock**	
	**hhs (1,214^1^)**		**hhs (495)**	
	***n***	**%**	***n***	**%**
<10%	746	61	381	77
10 to <20%	165	14	46	9
20 to <30%	91	7	24	5
30 to <40%	76	6	20	4
40 to <50%	43	4	11	2
≥50%	93	8	13	3

1 *No income data for one household*.

## Poultry Disease Risk and Losses

The “group” (herd/flock)-level disease prevalence tends to increase as size of the group increases, whereas within the “group” (herd/flock) prevalence of disease tends to decrease as group size increases [e.g., (12–14)]. The disease risk and losses were assessed separately for flocks of 1 to 20 birds (*n* = 1,037) and flocks of 21–50 birds (*n* = 178) at the time of data collection.

Table 4 displays the number and proportion of households reporting bird losses over the past year by cause for the two flock size groups. Disease was the most frequently reported cause of bird losses in both groups, having caused losses in around 60% of households. “Accident/injury,” including predation, was the second most frequent cause of bird losses (36 and 43%), followed by theft, which was experienced by around 20% of households. In both groups, the number of birds lost due to disease, accident or theft was markedly higher than the number of birds consumed, sold or gifted out, 10,512 vs. 5,133 and 2,370 vs. 1,435 in the smaller and larger flock-size groups, respectively.

**Table 4:** Number and proportion of households (hhs) experiencing bird losses and number of birds lost by cause in flocks of 1–20 and 21–50 birds (upper panel), and number, proportion and median value of birds lost by cause in flocks of 1–20 and 21–50 birds (lower panel).

	**1–20 birds (*****n*** **= 1,037 hhs)**			**21–50 birds (*****n*** **= 178 hhs)**		
**Households**	***n***	**%**	**Birds lost^1^**	***n***	**%**	**Birds lost^1^**
Disease	626	60.4	11.1/9	111	62.4	12.7/10
Accident/injury	373	36.0	7.3/5	76	42.7	9.7/6
Theft	174	16.8	4.9/3	32	18.0	7.1/6
**Birds**	***n***	**%**	**Mean bird value**	***n***	**%**	**Mean bird value**
Disease	6,940	66.0	3,533	1,408	59.4	3,866
Accident/injury	2,717	25.8	2,407	735	31.0	3,003
Theft	855	8.1	4,275	227	9.6	5,176
Total	10,512			2,370		

1 *Mean/median number of birds lost by households with losses*.

Disease was not only the most frequently experienced cause of bird losses, but with a median of 9 and 10 birds lost in flocks experiencing disease, disease was also responsible for the largest number and share of birds lost. Disease accounted for 66.0 and 59.4% of bird losses in the smaller and larger flocks size groups, respectively. The average value of birds lost to disease was intermediate between the value of stolen birds, which had the highest average value, and birds lost to accident/injury, and amounted to ~55% of the average value of birds sold.

Over a year, households with flocks of 1 to 20 birds on average lost 22% of their birds to disease (deaths/initial flock plus entries) with disease losses ranging from 0 to 93%. In the larger flock size group, average disease losses amounted to 13% of birds with a range of 0–59%. Table 5 displays the frequency distribution of the proportion of birds lost to disease for the two flock size groups.

**Table 5:** Frequency distribution of the proportion of birds lost to disease for flocks of 1–20 and of 21–50 birds.

**Share^1^ of birds lost to disease**	**1–20 birds**			**21–50 birds**		
	***n***	**%**	**Cumulative %**	***n***	**%**	**Cumulative %**
<10%	444	42.8	42.8	87	48.9	48.9
10 to <20%	105	10.1	52.9	44	24.7	73.6
20 to <30%	124	12.0	64.9	22	12.4	86.0
30 to <40%	124	12.0	76.9	15	8.4	94.4
40 to <50%	87	8.4	85.2	8	4.5	98.9
50 to <60%	62	6.0	91.2	2	1.1	100.0
60 to <70%	47	4.5	95.8	0	0.0	100.0
70 to <80%	31	3.0	98.7	0	0.0	100.0
80 to <90%	9	0.9	99.6	0	0.0	100.0
≥90%	4	0.4	100.0	0	0.0	100.0

1 *Number lost to disease over initial inventory plus entries*.

Larger flocks had a higher likelihood of sustaining a “small loss” (74 vs. 53% chance of losing <20% of the flock) but a lower risk of sustaining high loss, e.g., 1 vs. 15% risk of losing > 50% of the birds (Flock size itself apparently acts as “insurance” against “total loss” as it increases the likelihood of survivors.).

Newcastle disease was by far the disease most frequently mentioned to have affected poultry in both groups (53 and 55% of smaller and larger flocks) followed by fowl pox, reported by 3% of households with smaller flocks and 6% of households with larger flocks. Reported Newcastle disease vaccination (which did not take the frequency of vaccination into account), however, did not affect the proportion of birds lost to disease with average losses of 24% in flocks that had vaccinated and 21% in non-vaccinated flocks. Introduction of birds through purchase or gifts also did not affect the magnitude of disease losses, with average disease losses of 17% in flocks, which had introduced birds vs. average losses of 22% in flocks that had not introduced birds.

The value of birds lost to disease as proportion of total annual household income (Table 6) can serve as crude measure of economic impact on affected households. For more than half of all households, the value of birds lost to disease represented <1% of annual household income and for 95% of households it represented <10% of annual income. For a mere 2% of households, bird losses from disease represented more than 20% of annual household income.

**Table 6:** Frequency distribution of value of birds lost to disease as proportion of annual household income for flocks of 1–20 and of 21–50 birds.

**Value of birds lost as share of household income**	**1–20 birds**			**21–50 birds**		
	***n***	**%**	**Cumulative %**	***n***	**%**	**Cumulative %**
<1%	640	61.7	61.7	101	56.7	56.7
1 to <2%	131	12.6	74.3	26	14.6	71.3
2 to <3%	67	6.5	80.8	13	7.3	78.7
3 to <4%	52	5.0	85.8	8	4.5	83.1
4 to <5%	28	2.7	88.5	11	6.2	89.3
5 to <10%	69	6.7	95.2	10	5.6	94.9
10 to <20%	32	3.1	98.3	5	2.8	97.8
20 to <50%	14	1.4	99.6	2	1.1	98.9
≥50%	4	0.4	100.0	2	1.1	100.0

**Figure 1:**
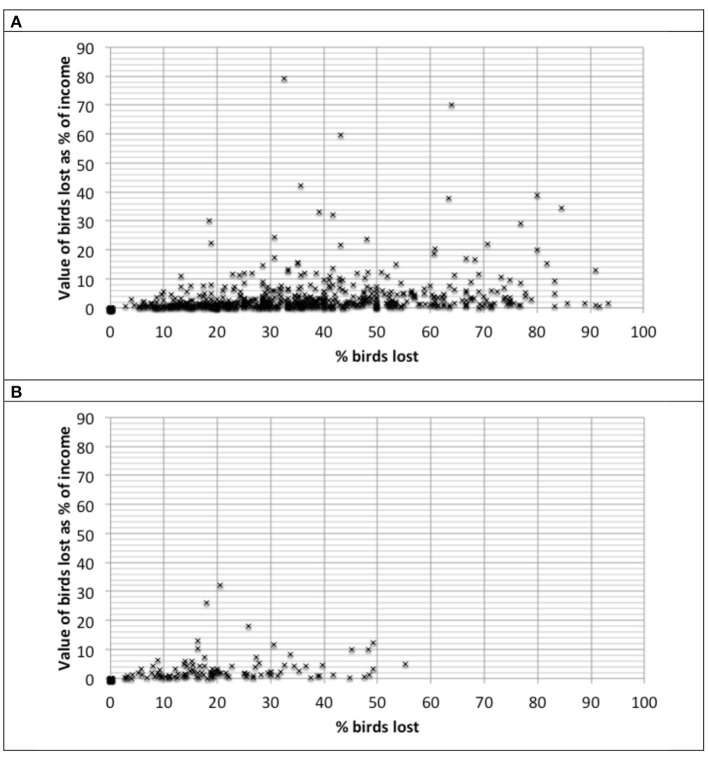
Relationship between the proportion of birds lost to disease and the value of lost birds as proportion of annual household income. **(A)** Flocks of 1–20 birds. **(B)** Flocks of 21–50 birds.

Figure 1 depicts the relationship between the proportion of birds lost to disease and the value of lost birds as proportion of annual household income. Clearly, the proportion of birds lost to disease is only a moderate predictor of the impact on total annual household income. A relatively small proportional loss of birds can translate into a relatively large loss in household income while conversely, a relatively high bird loss does not necessarily equate with a high loss in household income.

## Break-Even Cost of Biosecurity Investments

A partial budget analysis of the break-even cost of biosecurity investments leading to a reduction of disease losses by 10, 25, 70, and 75% was carried out for flocks of 1–20 and 21–50 birds. Average parameter values (e.g., initial and final inventory, number of birds lost to disease, number of eggs produced, etc.) and prices of the flock size groups of 1–20 and 21–50 birds were used for the analysis. The number of avoided egg losses from reduced disease mortality was estimated as the product of the number of deaths avoided and half of the average number of eggs produced per bird per year. The analysis does not assign a salvage value to diseased/dying birds, although these are often consumed. Details of the calculations are provided in the Annex.

The annual value of avoided bird and egg losses resulting from the maximum assessed disease reduction of 75% amounts to 24,682 TZ Sh. (app. USD 15.7) and 29,025 TZ Sh. (USD 18.5) for the smaller larger flock size groups, respectively (Table 7). These values thus represent the breakeven costs of biosecurity measures above which their cost would be higher than the returns. For both flock size groups, this reduction in disease-associated mortality would, if achieved at no cost, result in a one percent increase of annual household income.

**Table 7:** Household-level impacts of biosecurity interventions reducing the proportion of birds lost to disease by 10, 25, 50, and 75% in flocks of 1–20 and 21–50 birds.

	**Proportion of disease losses prevented**			
**Impact at household level**	**10%**	**25%**	**50%**	**75%**
**Flocks of 1–20 birds**				
Number of deaths avoided	0.67	1.67	3.35	5.02
Number of egg losses avoided	4.12	10.30	20.61	30.91
Value of avoided losses in TZ Sh.	3,291	8,227	16,455	24,682
Value of avoided losses in number of birds^1^	0.50	1.25	2.50	3.76
Increase in household income (%)	0.15	0.37	0.75	1.12
**Flocks of 21–50 birds**				
Number of deaths avoided	0.79	1.98	3.96	5.93
Number of egg losses avoided	3.31	8.28	16.55	24.83
Value of avoided losses in TZ Sh.	3,870	9,675	19,350	29,025
Value of avoided losses in number of birds^1^	0.56	1.40	2.80	4.19
Increase in household income (%)	0.13	0.32	0.64	0.96

1 *Having reached average age/weight for sale*.

Reducing disease associated mortality by 25%, a figure, which might be more realistic, would pay for itself if it could be achieved at a cost of around 8,000–10,000 TZ Sh. (USD 5.0–6.5) per year (around 0.5 USD/month).

## Discussion

In Tanzania, as elsewhere, the majority of rural poultry-keeping households have diversified income sources with cropping being the source of slightly over 50% of income, followed by non-agricultural activities contributing 35–40%. Poultry are generally managed as a low-input, low-output activity with minimal investments. More than three out of four households with poultry as their sole type of livestock obtain <10% of their annual income from poultry and are thus relatively resilient to shocks affecting their birds; although it is acknowledged that the loss of poultry may impact some household members more than others. However, around 10% of households with poultry as their sole type of livestock obtain 30% or more of their income from poultry, which makes them highly vulnerable to poultry disease and other events that decimate their flock.

Infectious disease is the most frequently reported cause of poultry losses (>60% of birds lost), resulting in an average loss of 22 and 13% of birds in smaller (1–20 birds) and larger (21–50 birds) flocks, respectively. In both flock size groups, disease attributable mortality translates into the loss of 7–8 birds per year per flock, more than the number of birds consumed and sold. Given this high toll of disease losses, efforts to reduce disease incidence appear highly warranted. However, considering the resource limitations (including labor) and diversified livelihoods strategies of village poultry keeping households, interventions need to be low-cost, highly effective and simple to implement.

No estimate of the reduction in disease risk is available for any of the biosecurity recommendations compiled by Conan et al. (5), either as stand-alone or as part of a combination of measures. Of the biosecurity recommendations, only indoor raising reduces (but does not necessarily eliminate) contact with wild birds, probably an important source of pathogen introduction. Indoor raising, however, comes at a high cost as it requires investment in a chicken house, additional expenses for chicken feed and additional labor to feed chicken and maintain cleanliness of the chicken house. In Cambodia for instance, the cost of building a poultry house is USD 25 when monthly family income is USD 75 (15). Using computer simulation to assess the cost and benefits of various forms of backyard poultry keeping, Gyeltshen et al. (16) found that housing had the maximum positive effect on flock size but resulted in net loss to the farmers. In addition to the cost of infrastructure, any biosecurity measure that restricts scavenging activities is associated with extra costs (and labor) of feeding the birds.

As scavenging is an essential element of village poultry keeping, the ceiling of achievable biosecurity through means other than housing may be low. In fact, in a cluster randomized trial of the impact of biosecurity measures (cleaning yards and equipment, quarantine of newly introduced and sick animals and burning dead birds) on poultry health in backyard flocks, Conan et al. (15) find that “*despite good compliance among poultry owners, the biosecurity intervention implemented in this study was not associated with improvements in poultry mortality rates. These findings suggest that basic biosecurity measures may not suffice to limit the spread of infectious diseases in backyard poultry flocks in Cambodia*.” Even for small-scale intensified poultry producers with housed poultry, the biosecurity “ceiling” is low as for most producers closed housing is prohibitively expensive (a closed house, which requires forced ventilation, costs about seven times as much as the prevailing “open” house) while open houses are not particularly biosecure.

Although improving biosecurity is likely to enhance flock productivity, a significant proportion of poultry keepers continue their “risky” production practices despite receiving advice on risk-reducing measures (17). Aini (18) attributed the low adoption of biosecurity measures by backyard farmers to their low cost-effectiveness.

Cost-effective measures to improve productivity of village poultry do exist, but these do not necessarily (exclusively) focus on disease risk. Predation of young chicks can be a significant cause of bird losses, exceeding the number of birds lost to disease [e.g., (19, 20)]. Henning et al. (21) assessed the impact of two interventions to improve backyard poultry production in Myanmar, namely (1) vaccination of individual birds against Newcastle disease (ND) and (2) improved management of chick rearing by providing coops for the protection of chicks from predation and chick starter feed. The benefit:cost ratio (BCR) for ND vaccination was very high (28.8) while the BCR for improved chick management was lower (4.7) but still high. Discounted Net Present Values for ND vaccination and improved chick management over a 10-year period were 30,791 and 167,825 Kyat (around 31 and 168 USD), respectively. Thus, despite high BCRs, the absolute benefits accruing from cost-effective improvements in backyard poultry production under Southeast Asian conditions appear rather moderate (0.26 and 1.4 USD/month). Research investments in the effective control of ND in village chickens via vaccination in sub-Saharan Africa has been calculated to yield a high BCR (22) with ongoing benefits where cost-sharing with farmers supports regular vaccination of village chickens against ND (23, 24).

Investments in poultry production with a high benefit:cost ratio may still prove to be unattractive to the average village poultry keeper as the additional return only results in a very modest increase in total household income and returns to investments in activities more central to their livelihood are possibly larger. Given the “livelihoods” impact of poultry disease losses is to a large extent determined by the share of household income derived from poultry, households relying heavily on poultry should be the ones most likely to adopt measures to mitigate poultry disease risks to improve production. Many of these households will be among the poorer in their respective communities and proposed interventions to improve poultry production need to be tailored to their specific circumstances, needs and capabilities rather than dwell on generic principles. The value placed on poultry within households may vary by gender (25) and unfortunately much of the data available to date does not allow analyses to be disaggregated by gender. It should also not be assumed, that disease is the most pressing problem and a more holistic, participatory and gender-sensitive approach to poultry production appears warranted. To appropriately tailor biosecurity and husbandry interventions to local conditions, it is essential that the various members (i.e., men, women and those of differing socio-economic and language groupings) of communities and households knowledgeable about poultry production be involved from the outset (26).

Disease risks to extensive rural poultry production have increased in many LMICs over the past two decades in association with the increased movement of intensively raised commercial poultry into rural areas. For example, the sale of spent hens in South Africa has been documented to contribute to the spread of ND in rural areas (27). Commercial birds that have been vaccinated against diseases such as ND, may display no clinical disease while shedding ND virus that can infect susceptible birds.

Developing biosecurity improvements for village poultry, considered worthwhile from a private farmer perspective, requires time for trust to be built and positive impacts achieved, including improved food safety and reduced zoonotic disease risks (28). Therefore, projects and programs must build in appropriate time and resources to support participatory approaches. Where risks of disease spread impact on the wider community and generate significant externalities, action should be taken, but efforts must be carefully targeted and poultry keepers supported by wider societal actions rather than being expected to invest in biosecurity for purely personal gain.

## Data Availability Statement

Publicly available datasets were analyzed in this study. This data can be found here: https://microdata.worldbank.org/index.php/catalog/2252/related-materials.

## Ethics Statement

Ethical review and approval was not required for the animal study because the data used for the analysis were secondary data.

## Author Contributions

JO has led the conceptual development, the data analysis, and the drafting of the initial manuscript. JR, RA, and ER contributed to the conceptual development and the drafting of the manuscript. All authors contributed to the article and approved the submitted version.

## Conflict of Interest

The authors declare that the research was conducted in the absence of any commercial or financial relationships that could be construed as a potential conflict of interest.

## Footnotes

^1^Livestock – animals kept for production, investment and sale, companion animals for pleasure and sporting animals.
