# Supplementary material for: Biosecurity in Village and Other Free-Range Poultry—Trying to Square the Circle?
Source: Front Vet Sci. 2021 Jun 2;8:678419. doi: 10.3389/fvets.2021.678419 (PMC8207203; doi:10.3389/fvets.2021.678419)
Supplement: Supplementary file 1 [file Table_1.docx]

**Biosecurity in village and other free-range poultry – trying to square the circle?**

**Joachim Otte^1^, Jonathan Rushton^2^, Elpidius Rukambile^3^, Robyn G. Alders^4,5^**

^1^ Berkeley Economic Advising and Research, California, USA

^2^ Institute of Infection, Veterinary and Ecological Sciences, University of Liverpool, UK

^3^ Tanzania Veterinary Laboratory Agency, Dar es Salaam, Tanzania

^4^ Kyeema Foundation, Brisbane, Australia
^5^ Development Policy Centre, Australian National University, Canberra, Australia

*** Correspondence:**

Jonathan Rushton

jrushton@liverpool.ac.uk

**ANNEX**

**Table A1**: Calculation of avoided bird and egg losses by reduction of disease associated mortality by 10, 25, 50 and 75% in flocks of 1 to 20 birds

|  |  | **Population** | **/Hh Flock** | **/Bird (Av)** |
| --- | --- | --- | --- | --- |
|  | Households | 1,037 |  |  |
|  | HH income | 2,280,548,499 | 2,199,179 |  |
|  | Start inventory | 9,236 | 8.91 |  |
|  | End inventory | 9,461 | 9.12 |  |
| **Entries** | Hatched in | 14,989 | 14.45 | 1.60 |
|  | Purchase | 787 | 0.76 | 0.08 |
|  | Gift in/payment | 94 | 0.09 | 0.01 |
| **Exits** | Disease | 6,940 | 6.69 | 0.74 |
|  | Accident | 2,717 | 2.62 | 0.29 |
|  | Theft | 855 | 0.82 | 0.09 |
|  | Consumption | 2,702 | 2.61 | 0.29 |
|  | Sale | 2,057 | 1.98 | 0.22 |
|  | Gift out/payment | 374 | 0.36 | 0.04 |
| **Eggs** | Home use | 109,010 | 105.12 | 11.66 |
|  | Sale | 6,127 | 5.91 | 0.66 |
|  | Total | 115,137 | 111.03 | 12.32 |
| **Values** | Birds: consumption, sale, gift | 33,727,366 | 32,524 | 3,608 |
|  | Inventory change | 795,016 | 767 | 85 |
|  | Eggs: Home use, sale | 25,877,168 | 24,954 | 2,768 |
|  | Total | 60,399,549 | 58,245 | 6,461 |
|  | **Prop. Disease Prevented (%)** | **10.00** |  |  |
|  | Nr deaths avoided | 694 | 0.67 | 0.07 |
|  | Nr of avoided egg losses | 4,274 | 4.12 | 0.46 |
|  | Value of avoided losses - birds | 2,452,181 | 2,365 | 262 |
|  | Value of avoided losses - eggs | 960,515 | 926 | 103 |
|  | Total | 3,412,696 | 3,291 | 365.05 |
|  | Value as nr of birds for sale | 519 | 0.50 | 0.06 |
|  | Increase in hh income (%) | 0.15 | 0.15 |  |
|  | **Prop. Disease Prevented (%)** | **25.00** |  |  |
|  | Nr deaths avoided | 1,735 | 1.67 | 0.19 |
|  | Nr of avoided egg losses | 10,684 | 10.30 | 1.14 |
|  | Value of avoided losses - birds | 6,130,453 | 5912 | 656 |
|  | Value of avoided losses - eggs | 2,401,288 | 2316 | 257 |
|  | Total | 8,531,741 | 8,227 | 913 |
|  | Value as nr of birds for sale | 1,298 | 1.25 | 0.14 |
|  | Increase in hh income (%) | 0.37 | 0.37 |  |
|  | **Prop. Disease Prevented (%)** | **50.00** |  |  |
|  | Nr deaths avoided | 3,470 | 3.35 | 0.37 |
|  | Nr of avoided egg losses | 21,368 | 20.61 | 2.29 |
|  | Value of avoided losses - birds | 12,260,906 | 11,823 | 1,312 |
|  | Value of avoided losses - eggs | 4,802,576 | 4,631 | 514 |
|  | Total | 17,063,482 | 16,455 | 1,825 |
|  | Value as nr of birds for sale | 2,597 | 2.50 | 0.28 |
|  | Increase in hh income (%) | 0.75 | 0.75 |  |
|  | **Prop. Disease Prevented (%)** | **75.00** |  |  |
|  | Nr deaths avoided | 5,205 | 5.02 | 0.56 |
|  | Nr of avoided egg losses | 32,053 | 30.91 | 3.43 |
|  | Value of avoided losses - birds | 18,391,359 | 17,735 | 1,967 |
|  | Value of avoided losses - eggs | 7,203,865 | 6,947 | 771 |
|  | Total | 25,595,224 | 24,682 | 2,737.90 |
|  | Value as nr of birds for sale | 3,895 | 3.76 | 0.42 |
|  | Increase in hh income (%) | 1.12 | 1.12 |  |

**Table A2**: Calculation of avoided bird and egg losses by reduction of disease associated mortality by 10, 25, 50 and 75% in flocks of 21 to 50 birds

|  |  | **Population** | **/Hh Flock** | **/Bird (Av)** |
| --- | --- | --- | --- | --- |
|  | Households | 178 |  |  |
|  | HH income | 537,306,035 | 3,018,573 |  |
|  | Start inventory | 4,497 | 25.26 |  |
|  | End inventory | 5,602 | 31.47 |  |
| **Entries** | Hatched in | 4,754 | 26.71 | 0.94 |
|  | Purchase | 143 | 0.80 | 0.03 |
|  | Gift in/payment | 13 | 0.07 | 0.00 |
| **Exits** | Disease | 1,408 | 7.91 | 0.28 |
|  | Accident | 735 | 4.13 | 0.15 |
|  | Theft | 227 | 1.28 | 0.04 |
|  | Consumption | 775 | 4.35 | 0.15 |
|  | Sale | 555 | 3.12 | 0.11 |
|  | Gift out/payment | 105 | 0.59 | 0.02 |
| **Eggs** | Home use | 40,414 | 227.04 | 8.00 |
|  | Sale | 1,853 | 10.41 | 0.37 |
|  | Total | 42,267 | 237.46 | 8.37 |
| **Values** | Birds: consumption, sale, gift | 40,414 | 227.04 | 8.00 |
|  | Inventory change | 1,853 | 10.41 | 0.37 |
|  | Eggs: Cons, sale | 42,267 | 237.46 | 8.37 |
|  | Total | 40,414 | 227.04 | 8.00 |
|  | **Prop. Disease Prevented (%)** | **10.00** |  |  |
|  | Nr deaths avoided | 141 | 0.79 | 0.03 |
|  | Nr of avoided egg losses | 589 | 3.31 | 0.12 |
|  | Value of avoided losses - birds | 544,380 | 3,058 | 108 |
|  | Value of avoided losses - eggs | 144,475 | 812 | 29 |
|  | Total | 688,855 | 3,870 | 136.42 |
|  | Value as nr of birds for sale | 100 | 0.56 | 0.02 |
|  | Increase in hh income (%) | 0.13 | 0.13 |  |
|  | **Prop. Disease Prevented (%)** | **25.00** |  |  |
|  | Nr deaths avoided | 352 | 1.98 | 0.07 |
|  | Nr of avoided egg losses | 1,473 | 8.28 | 0.29 |
|  | Value of avoided losses - birds | 1,360,950 | 7,646 | 270 |
|  | Value of avoided losses - eggs | 361,188 | 2,029 | 72 |
|  | Total | 1,722,138 | 9,675 | 341 |
|  | Value as nr of birds for sale | 249 | 1.40 | 0.05 |
|  | Increase in hh income (%) | 0.32 | 0.32 |  |
|  | **Prop. Disease Prevented (%)** | **50.00** |  |  |
|  | Nr deaths avoided | 704 | 3.96 | 0.14 |
|  | Nr of avoided egg losses | 2,946 | 16.55 | 0.58 |
|  | Value of avoided losses - birds | 2,721,900 | 15,292 | 539 |
|  | Value of avoided losses - eggs | 722,376 | 4,058 | 143 |
|  | Total | 3,444,276 | 19,350 | 682 |
|  | Value as nr of birds for sale | 498 | 2.80 | 0.10 |
|  | Increase in hh income (%) | 0.64 | 0.64 |  |
|  | **Prop. Disease Prevented (%)** | **75.00** |  |  |
|  | Nr deaths avoided | 1,056 | 5.93 | 0.21 |
|  | Nr of avoided egg losses | 4,420 | 24.83 | 0.88 |
|  | Value of avoided losses - birds | 4,082,850 | 22,937 | 809 |
|  | Value of avoided losses - eggs | 1,083,563 | 6,087 | 215 |
|  | Total | 5,166,413 | 29,025 | 1,023 |
|  | Value as nr of birds for sale | 746 | 4.19 | 0.15 |
|  | Increase in hh income (%) | 0.96 | 0.96 |  |

- Value of avoided losses – birds: number x average value of dead bird reported for group
- Value of avoided losses – eggs: number of avoided deaths x average number of eggs per bird/2 x average value of egg reported for group
